# Results of Surgical Treatment of Occult Spinal Dysraphisms—A Single Centre Experience

**DOI:** 10.3390/diagnostics14070703

**Published:** 2024-03-27

**Authors:** Peter Spazzapan, Tomaz Velnar, Nina Perosa, Andrej Porcnik, Borut Prestor

**Affiliations:** 1 Unit of Paediatric Neurosurgery, University Medical Centre Ljubljana, 1000 Ljubljana, Slovenia; spazzapanpeter@yahoo.it; 2 Department of Neurosurgery, University Medical Centre Ljubljana, 1000 Ljubljana, Slovenia; nina.vrbnjak@gmail.com (N.P.); andrej.porcnik@kclj.si (A.P.); borut.prestor@kclj.si (B.P.)

**Keywords:** dysraphisms, tethered cord, sphincter dysfunction, paresis, pain, orthopaedic deformation

## Abstract

Occult spinal dysraphisms (OSDs) are caused by various defects in the embryogenesis of the spinal cord and represent an obstacle to the ascent of the conus, which allows the conus to pass from the lower levels of the spinal canal to the final position between L1 and L2 during normal foetal life. When an OSD tethers the spinal cord at the lower levels, it can lead to neurological symptoms, better known as tethered cord syndrome. Surgical treatment of OSD is primarily aimed at untethering the spinal cord. In asymptomatic patients, this can protect against the long-term development of neurological deficits. In symptomatic patients, this can halt or limit the progression of existing symptoms. The aim of this study is to examine all paediatric and adult patients diagnosed with OSD and treated in the Department of Neurosurgery at the University Medical Centre Ljubljana during the 5-year period of 2016–2021. All patients diagnosed with OSD during this period were included in the study. Patient characteristics, treatment modalities and outcomes were studied with the aim of describing the differences between the paediatric and adult population and defining the rationality of treating these pathological conditions. We included in the study 52 patients with 64 occult dysraphic lesions. Adults (>18 years old) represented 15/52 (28.8%) of all patients, while 37/52 (71.8%) were children. The most common OSDs were conus lipomas, followed by dermal sinus tracts, filum terminale lipomas and split cord malformations. Surgical treatment was performed in 35/52 (67.3%) cases, while conservative management was chosen in 17/52 (32.6%) cases. The preoperative presence of symptoms was statistically higher in adults than in children (*p* = 0.0098). Surgery on complex spinal cord lipomas was statistically related to a higher rate of postoperative neurological complications (*p* = 0.0002). The treatment of OSD is complex and must be based on knowledge of the developmental anomalies of the spine and spinal cord. Successful surgical treatment relies on microsurgical techniques and the use of neuromonitoring. Successful treatment can prevent or limit the occurrence of neurological problems.

## 1. Introduction

Occult spinal dysraphisms (OSDs) are malformations of the spinal axis of the nervous system, caused by disorders at different stages of the embryogenesis of the nervous system. Depending on the developmental stage at which the defect occurs, they are categorised as gastrulation defects, primary neurulation defects and secondary neurulation defects, which are described as follows:

− Gastrulation defects: Gastrulation is the process by which the epiblast gives rise to the trilaminar disc (ectoderm, mesoderm and endoderm). Defects at this stage can lead to the development of diastematomyelia (split cord malformation—SCM) and neurenteric cysts.
− Primary neurulation defects: Primary neurulation is the process of neural tube closure. A defect at this stage can lead to the development of a myelomeningocele (MMC) (open spinal dysraphism, not discussed in this article), dermal sinus tract, limited dorsal myeloschisis (LDM) and conus lipoma.
− Secondary neurulation defects: Secondary neurulation is the process of formation of the most caudal part of the nervous system (conus, cauda equina and filum terminale). Defects at this stage lead to the development of lipomas of the filum terminale (LFTs), Currarino syndrome, caudal regression syndrome and myelocystocele.

One of the key moments in the development of the spinal cord and cauda equina is the ascent of the conus, i.e., the process by which the conus ascends from the low, sacral levels to the final L1–L2 position, during the last months of gestation. All forms of OSD can interfere with this process to some degree, by impeding the ascent of the conus or spinal cord, which remains tethered in the lower levels of the spinal canal. This condition is called tethered spinal cord. A tethered spinal cord may be asymptomatic in early childhood. Later, during growth, some children may develop tethered cord syndrome, which is characterised by orthopaedic deformities, paresis, muscle atrophy, paraesthesia, pain and sphincter dysfunction. Asymptomatic children with tethering of the spinal cord have a varying risk of becoming symptomatic in the first 10 years of life, depending on the type of OSD. For conus lipomas, which are one of the most common forms of OSD, this risk is estimated at 40–43% [1,2,3]. Some children with OSD may already show symptoms in the neonatal period, though cutaneous stigmata in the lumbosacral region remain the most common cause that induce further investigations and lead to the diagnosis of OSD [1,2]. In all cases of OSD, it must be assessed whether it is appropriate to surgically untether the spinal cord to prevent the onset of symptoms and to prevent the progression of pre-existing neurological deficits.

In this study we analysed the preoperative, operative and postoperative data of a series of consecutive patients treated in a single centre for OSD. The aim was to compare the preoperative data between adults and children and define the operative risks of different types of OSDs.

## 2. Materials and Methods

In this paper, we retrospectively review all preoperative and postoperative data of patients treated with newly diagnosed OSD in the Department of Neurosurgery at the University Medical Centre Ljubljana between 2016 and 2021.

We collected data on the demographic, clinical and radiological characteristics of the patients. Lipomas of the spinal cord were classified, based on the definition of Pang [3], as dorsal (located on the dorsal side of the placode and above the conus, which is spared), transitional (located on the dorsal side of the placode, but at the level of the conus, which is affected) and chaotic (located on the ventral and dorsal side of the conus, which is embedded in the fatty tissue). The treatment was divided into conservative or surgical and the outcome of treatment was classified on the basis of new postoperative neurological deficits. To compare the differences between paediatric and adult patients we used a Student’s *t*-test for two independent means. We further analysed the operative risks for those subtypes of OSD related to the highest surgical challenges and risks, namely the transitional and chaotic conus lipomas. The statistical results were significant, at *p* < 0.05.

## 3. Results

Between 2016 and 2021, 52 patients were diagnosed with some form of OSD. Table 1 summarises the demographic, clinical and radiological aspects of the patients (Table 1). Adults (>18 years old) represented 15/52 (28.8%) of all patients, while 37/52 (71.8%) were children. Of paediatric diagnoses, 21/37 (56.7%) were made within the first 3 months of life. Cutaneous stigmata (haemangioma, subcutaneous lipoma or dermal pit) were present in 44/52 (84.6%) patients; they were recognized in 34/37 (91.8%) paediatric patients and in 10/15 (66.6%) adult patients.

**Figure 1:**
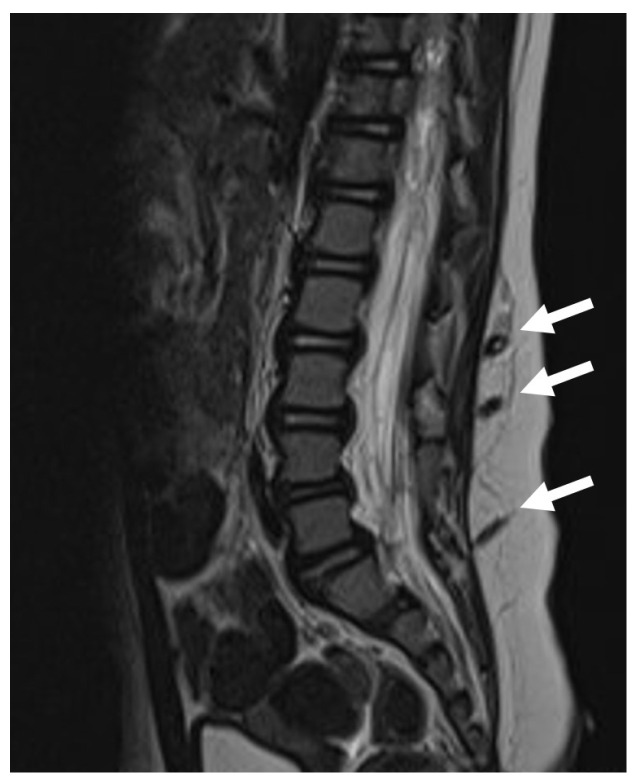
MR scan of a paediatric patient with three concomitant dermal tracts (white arrows). The conus is located at a physiological level L1.

In 13/52 (25%) cases there were two different dysraphic lesions. Two patients had three different dysraphic lesions (Figure 1). In total, there were 64 dysraphic lesions (Table 1): 22/64 (34.3%) conus lipomas (transitional in 13/22 (59%) cases, dorsal in 7/22 (31.8%) cases and chaotic in 2/22 (9%) cases); 13/64 (20.3%) dermal tracts; 12/64 (18.7%) LFTs; 5/64 (7.8%) SCMs; 3/64 (4.6%) agenesis of the conus as part of caudal regression syndrome; 2/64 (3.1%) LDM; 2/64 (3.1%) dermoid cysts; 2/64 (3.1%) presacral meningoceles as part of Currarino syndrome; 1/64 (1.5%) neurenteric cyst; 1/64 (1.5%) epithelialized MMC; and 1/64 (1.5%) meningocele.

**Figure 2:**
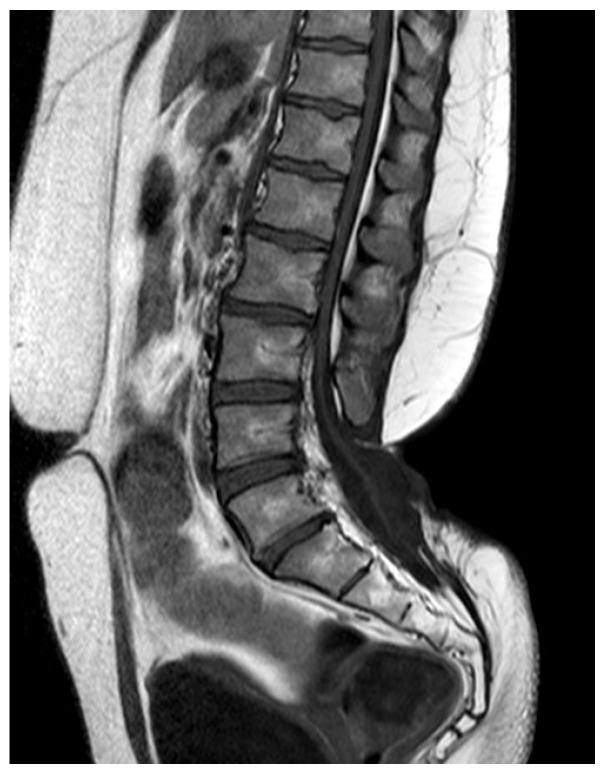
MR scan of an adult patient who presented with an epithelized MMC tethering the spinal cord. The spinal cord was untethered and the dural sac reconstructed by means of a wide dural sac.

The only case of epithelialized MMC (Figure 2) was included in the OSD series because the patient presented in adulthood and the placode was completely covered with intact skin. The spinal cord was untethered from the overlying fibrous epithelialized tissue, and the dural sac was reconstructed using the same techniques and principles of untethering for spinal cord lipomas.

**Figure 3:**
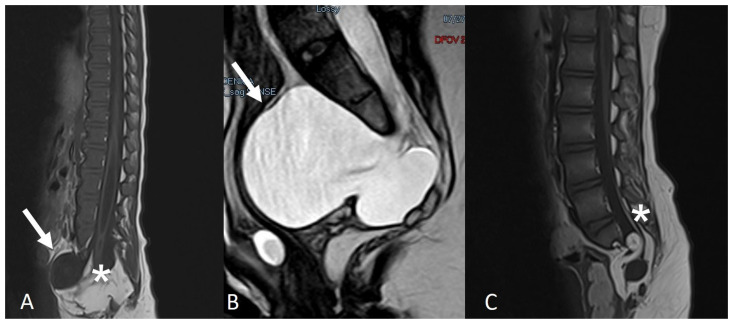
Two patients with Currarino syndrome, characterised by a presacral mass, which is most often represented by a meningocele (**A**,**B**) (white arrows). The spinal cord can be tethered by a conus lipoma (**A**) or by a LFT (**C**) (asterisks).

**Figure 4:**
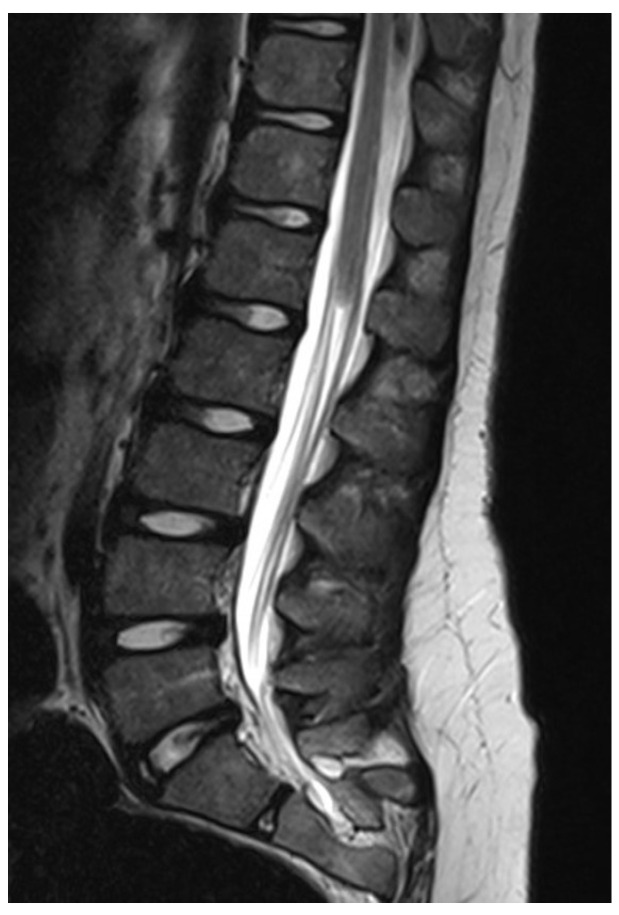
A case of caudal regression syndrome, where the conus, cauda equina and filum terminale are not developed, due to an absent process of secondary neurulation.

In 8/52 (15.3%) cases, the conus was at the physiological level of L1, while, in all other cases, the spinal cord was tethered at lower levels. The VACTERL association (combination of spinal defects, anal atresia, cardiac defects, tracheoesophageal fistula, renal anomalies and orthopaedic deformities of the lower limbs) was present in 2/52 (3.8%) cases. Genetic syndromes were present as follows: Currarino syndrome in 3/52 (5.7%) cases (Figure 3), caudal regression syndrome in 3/52 (5.7%) cases (Figure 4) and sacral syndrome, Down syndrome and PNET hamartoma syndrome each in 1/52 (1.9%) cases. A Chiari malformation was present in 4/52 (7.6%) cases (Chiari 2 in two cases with an underlying MMC). The two cases in which the primary underlying malformation was an open neural tube defect, namely an MMC, had also associated hydrocephalus, which were treated by means of a ventriculoperitoneal shunt. Overall, 17/52 (32.6%) patients were not treated surgically, and 35/52 (67.3%) patients were treated with a neurosurgical procedure (Table 2).

The reasons for not undergoing surgery were as follows: rejection of the risks of surgery or an older age of the patient (the preventive potential of surgical untethering is lower in adults than in children) [1,2,3]. In three cases with caudal regression syndrome, no surgery was indicated, as magnetic resonance imaging (MRI) showed only agenesis of the conus without spinal cord tethering.

In the preoperative neurological status, we observed the following (Table 3): spinal deformities in 5/37 (13.5%) paediatric cases and in 3/15 (20%) adult cases (*p* = 0.566); urological problems in 7/37 (18.9%) paediatric cases and 5/15 (33.3%) adult cases (*p* = 0.272); orthopaedic deformities in 9/37 (24.3%) paediatric cases and 8/15 (53.3%) adult cases (*p* = 0.044); lower limb paresis in 8/37 (21.6%) paediatric cases and 7/15 (46.6%) adult cases (*p* = 0.073); pain in 5/37 (13.5%) paediatric cases and 11/15 (73.3%) adult cases (*p* = 0.0001); and paraesthesia in 10/37 (27%) paediatric cases and 10/15 (66.6%) adult cases (*p* = 0.007). Overall, the statistical analysis confirmed a statistically significant difference in the presence of symptoms between children and adults (*p* = 0.0098).

Syringomyelia was present in 12/37 (32.4%) paediatric cases and in none of the adult cases. In four paediatric cases (three caudal regression syndromes and one MMC), the primary spinal cord malformation, rather than the spinal cord tethering, was the direct cause of the neurological deficits. In the group of symptomatic children, no subtype of dysraphism was predominant (three LFTs, four conus lipomas, two SCMs and three caudal regression syndromes).

Neurosurgical untethering of the spinal cord was performed in 35/52 (67.3%) cases. Postoperative transient sphincter dysfunction occurred in 2/35 (5.7%) cases and permanent sphincter dysfunction occurred in 1/35 (2.8%) case. In 3/35 (8.5%) cases, a permanent loss of sensation was observed in some isolated dermatomes of the lower limbs after the untethering. All of these complications occurred after transitional conus lipoma surgery. Wound infection occurred in 2/35 (5.7%) cases, while we observed cerebrospinal fluid (CSF) leakage in 3/35 (8.5%) cases. A second operation was not necessary in any case. Taking into account the complexity of surgical treatment of transitional and chaotic lipomas, we observed that 4/9 (44.4%) of these surgeries were complicated by a permanent neurological deficit, compared to the 0% rate of permanent neurological deficits of the other 26 OSD surgical procedures (*p* = 0.0002).

## 4. Discussion

OSDs are developmental anomalies of the spinal neural axis that can cause tethered cord syndrome. In the last trimester of pregnancy, when the nervous system is already developed, the physiological process of the ascent of the conus takes place. Due to the asymmetry between the development and growth of the vertebrae and the spinal cord, the conus ascents from its original low position to the physiological level of L1–L2. In the vast majority of cases, OSD interferes with this process and tether the spinal cord at a low level. In the long term, this leads to ischemia, compression and myelodysplasia of the spinal cord, which can result in tethered cord syndrome, characterized by spinal and orthopaedic deformities, urological problems, paresis, paraesthesia and pain in the lower limbs.

OSDs are caused by a variety of early embryological neurodevelopmental defects. The most appropriate classification of dysraphisms is, therefore, based on knowledge of the developmental stages of embryonic development.

### 4.1. Defects in the Gastrulation Stage

Gastrulation occurs between days 14 and 16 of gestational age and leads to the development of the trilaminar embryonic disc, which consists of the endoderm, mesoderm and ectoderm. The ectoderm is the most important from the point of view of neurosurgical pathology.

**Figure 5:**
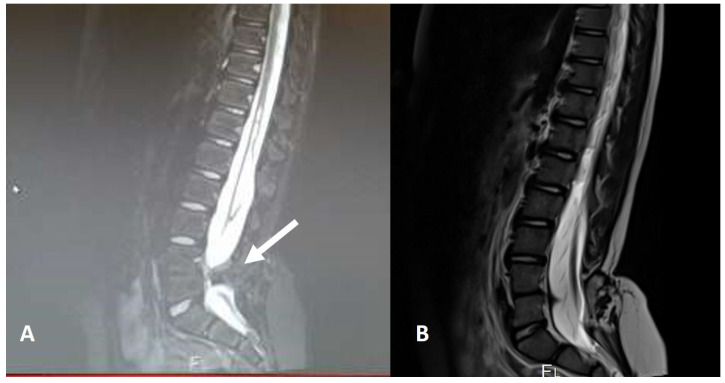
A preoperative (**A**) scan of a SCM1 with a bone spur separating two dural sacs and two hemicords (white arrow). The postoperative image (**B**) shows the removal of the bone spur and the reconstruction of a single dural sac.

1. Diastematomyelia—Split cord malformation (SCM):

SCM is characterised by a split, double spinal cord. It is caused by a defect in the phase of cellular migration from the epiblast to the primitive cord, a process that normally leads to cellular integration and the formation of the notochord. If integration does not occur, two notochords are formed, triggering the formation of two hemicords [4]. A distinction is made between SCM1, in which there is a bone spur between the two halves of the spinal cord, and SCM2, in which the two hemicords are separated by a fibrous membrane. SCM1 is characterised by two dural sacs, while the dural sac in SCM2 is a single one. Both forms hinder the normal ascent of the conus and the spinal cord. For this reason, it is necessary to remove the bone or membrane separating the two hemicords and create a single dural sac. In our series, SCM1 was present in three cases and SCM2 in two cases. Two patients with SCM1 were treated surgically (Figure 5), and one patient with SCM1 rejected surgical treatment. Both patients with SCM2 are asymptomatic and are under strict follow-up with annual electromyographic and urodynamic tests.

**Figure 6:**
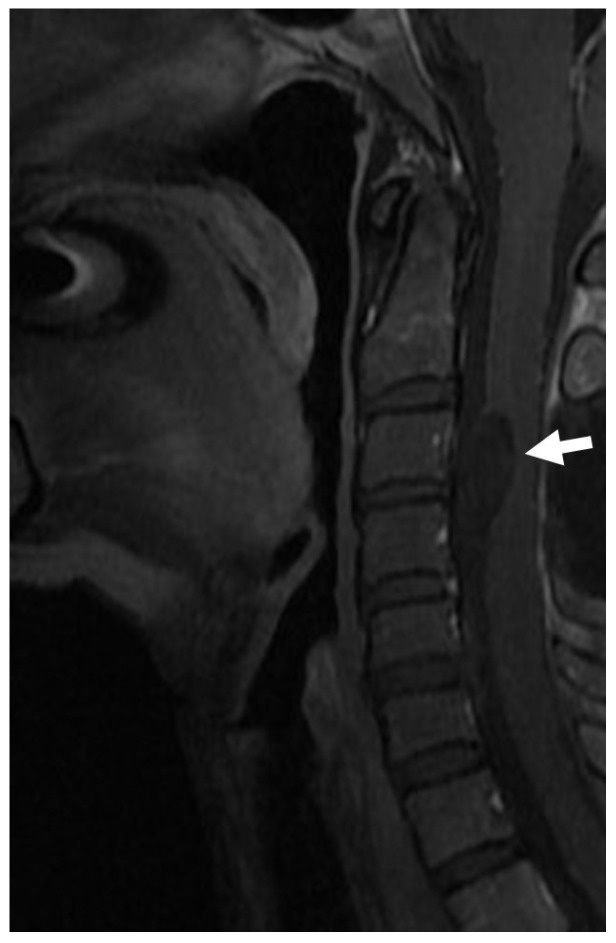
A neurenteric cyst located on the ventral side of the cervical spinal cord (white arrow). The patient presented in adulthood due to neurological symptoms related to the compression of the spinal cord.

2. Neurenteric cyst:

Between days 18 and 20 of gestation, the yolk sac and amniotic cavity are temporarily connected by the neurenteric canal of Kovalevsky, which remains open for 3–4 days and closes with the closure of the primitive streak, leaving the notochord behind [5]. If this channel does not close, this leads to a permanent connection between the ectodermal (neural) and endodermal structures. This form of OSD is called a neurenteric cyst, which manifests as a tumour in the spinal canal (Figure 6). The cyst is surrounded by a characteristic enteric epithelium and can compress the spinal cord, leading to neurological deficits. A neurenteric cyst is always located on the ventral or lateral side of the spinal cord, never on the dorsal side. It is often associated with bony abnormalities of the spine and is never located under the S2 neurotome, as the nervous system distal to this level develops through a separate process called secondary neurulation. The neurenteric cyst occurred in only one case in our series: it became symptomatic in adulthood due to compression of the spinal cord and was not associated with a definite bony abnormality of the spine. A neurenteric cyst must be removed microsurgically. Any associated bony malformations or deformities of the spine should be treated separately, if necessary, with spondylodesis. In our series, one adult patient presented with a neurenteric cyst at the level of C2–3, which was successfully removed through a posterior approach.

### 4.2. Defects in the Primary Neurulation Stage

Primary neurulation is a process that occurs between days 17 and 23 of gestational age, in which two neural crests within the ectoderm elevate and fuse to form the neural tube. Primary neurulation also includes the process of disjunction: this is the moment when the closed neural tube (neuroectoderm) separates from the overlying cutaneous ectoderm. From this moment on, the closed neural tube will be surrounded by cells of mesodermal origin.

The most common and dramatic defect that occurs at this stage of embryonic development is MMC, which results from incomplete closure of the neural tube. MMC is an open spinal dysraphism, in which the open neural tube is directly exposed to the outside. MMC is a malformation that affects the entire nervous system and leads to a Chiari 2 malformation, hydrocephalus and many other malformations of the central nervous system (polymicrogyria, agenesis of the vermis, agenesis of the corpus callosum, etc.). Since it is an open neural tube defect, it will not be discussed here.

The other forms of OSD that occur at the stage of primary neurulation are related to defects in the process of disjunction, which may be either incomplete or premature.

**Figure 7:**
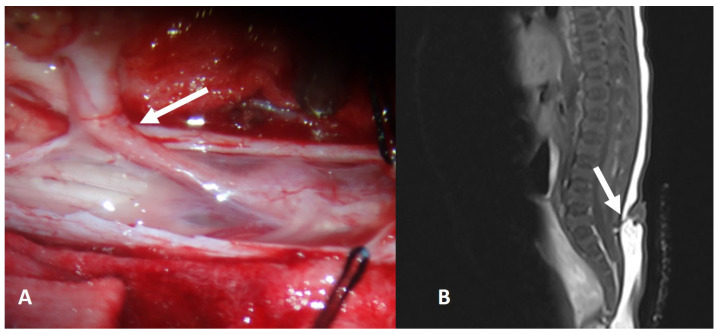
Intraoperative view (**A**) and MR scan (**B**) of a child with a lumbosacral dermal tract (white arrow) and an intradural dermoid cyst. The tract must be removed completely, as close as possible to the spinal cord.

1. Dermal tract and limited dorsal myeloschisis (LDM):

A dermal tract develops when the disjunction process is incomplete [6]. A dermal tract is a thin fibrous band that connects the skin (dermal ectoderm) to the central nervous system (neuroectoderm). The tract usually runs through the dysraphic dorsal elements of the spinal column and through the dura and attaches to the spinal cord or another intradural neural element. The dermal tract is often associated with an intradural inclusion cyst (dermoid or epidermoid) (Figure 7), which may compress the surrounding neural structures.

**Figure 8:**
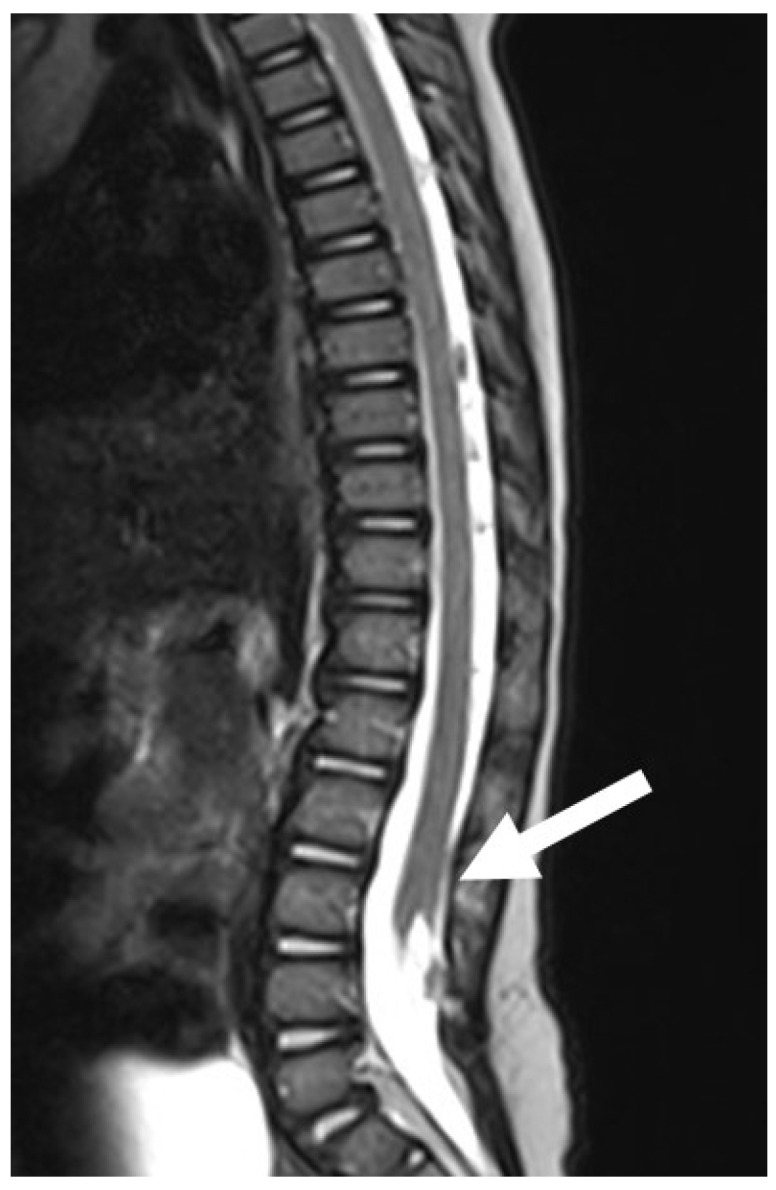
LDM is formed by neural tissue that is tethered toward the extraspinal mesodermal tissues. Note the deformed dorsal aspect of the spinal cord at the level of the dysraphic lesion (white arrow), which can be located at any level of the spinal cord.

If the histological composition of the fibrous tract is predominantly neural rather than fibrous, it is better defined as LDM (Figure 8) [7]. When there is communication between the CSF and the subcutaneous compartment through the neural tract, the LDM can take on a cystic morphology. In these cases, the skin overlying the subcutaneous cyst can be very thin, so these cases may be difficult to distinguish from MMC, especially on prenatal ultrasounds. Undoubtedly, the neurological and general prognosis is much worse in MMC than in LDM.

Dermal tracts were a fairly common finding in our series, accounting for 20.3% of all OSD. LDM was a rather rare diagnosis, at 3.1% of all OSD. Surgical removal of the dermal tract or LDM is necessary to relieve the spinal cord and prevent possible infection associated with the abnormal dermal and epidermal tissue. The aim of surgery should be to completely remove the dermal tract or LDM, as close to the spinal cord as possible, without causing neurological deficits. Neurophysiological monitoring is essential for these and all other OSD operations.

**Figure 9:**
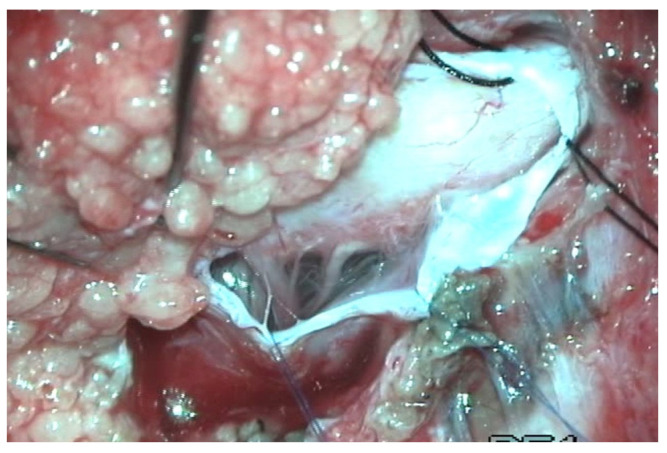
Intraoperative picture of a transitional conus lipoma, attached to the neural placode. The adipose tissue must be completely removed to guarantee a long-term protective effect against neurological deterioration.

**Figure 10:**
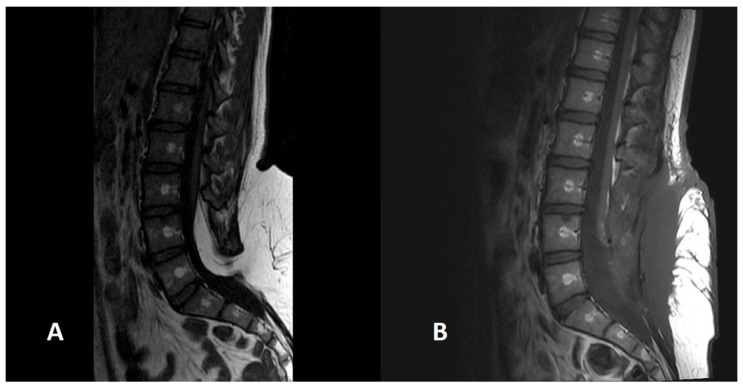
Preoperative (**A**) and postoperative (**B**) MR image of a completely removed transitional conus lipoma.

2. Lipoma of the conus:

Premature disjunction leads to the formation of spinal cord lipomas. If the neuroectoderm separates from the cutaneous ectoderm too early, when the neural tube has not yet closed, this leads to infiltration of the still open neural tube by mesodermal tissue. The neural tube can therefore not close, and the placode (the exposed neural plate) is infiltrated by subcutaneous fatty tissue (Figure 9). This more or less thick lipomatous stalk can migrate from the subcutaneous tissue through the dysraphic bone and the dura into the intradural space. Lipomas of the spinal cord can be of the dorsal type (located on the dorsal side of the spinal cord and cranial to the still recognisable conus), of the transitional type (located on the dorsal side of the spinal cord and at the level of the no longer recognisable conus) or of the chaotic type (located on the dorsal and ventral side of the spinal cord, with an unrecognisable conus). Surgery for spinal cord lipomas, especially of the transitional and chaotic type, is complex and risky. Without surgery, 40–43% of these children develop tethered cord syndrome within 10 years [1,2,8,9]. At the same time, partial resection is also not recommended, as the results of subtotal removal are even worse than those of conservative treatment (follow-up with electromyography and urodynamic examinations), with 46% of these children developing tethered cord syndrome [8,9]. It is therefore necessary to completely remove the fatty tissue, undertake neurulation of the placode and create a wide dural sac with extensive duraplasty. Intraoperatively, it is often difficult to recognize the border between fatty and neural tissue, which is why neurophysiological monitoring is an absolute priority in these procedures. Using this technique, the risk of new neurological deficits is reduced to 0.8% over 10 years [8]. It is important to note that the risk of neurological deficits and the long-term prognosis vary, according to the type of lipoma and the position of the conus. Transitional lipomas with a low-lying conus are at high risk, compared to dorsal lipomas where the conus may be located at L1, L2 or L3 level. The higher risk carried by transitional and chaotic lipomas was confirmed also by our results: adult patients, in whom neurological deficits were statistically significantly more frequent than in children (*p* = 0.0098), also had a higher incidence of transitional and chaotic lipomas at diagnosis compared to children (57.1% vs. 44.4%). In our series, 15/22 (68.1%) conus lipomas were surgically treated (Figure 10). Our results confirm that conus lipoma surgery is safe, although the complexity of treatment of transitional and chaotic lipomas was evident, since permanent neurological deficits (one neurogenic bladder and three dermatomal sensorial deficits) were present exclusively in four of nine (44.4%) patients who had a transitional or chaotic lipoma (*p* = 0.0002).

### 4.3. Defects in the Secondary Neurulation Stage

While the central nervous system, cranial to the S2 neurotome, is formed by the process of primary neurulation, a completely different process called secondary neurulation occurs caudal to this level. Around the 25th to 27th day of gestational age, a caudal cellular mass, consisting of undifferentiated pluripotent cells, forms in the caudal part of the embryo. These cells undergo an extraordinary proliferation phase, followed by a phase of apoptotic decline and regression of the caudal cell mass. This process gives rise to the conus, the filum terminale and the cauda equina. These tissues then connect to the caudal part of the neural tube that was formed by primary neurulation. The process of secondary neurulation is closely linked to the development of the cloaca. Failures at this stage can therefore lead to complex syndromes in which the nervous, urogenital and digestive organs are affected simultaneously.

**Figure 11:**
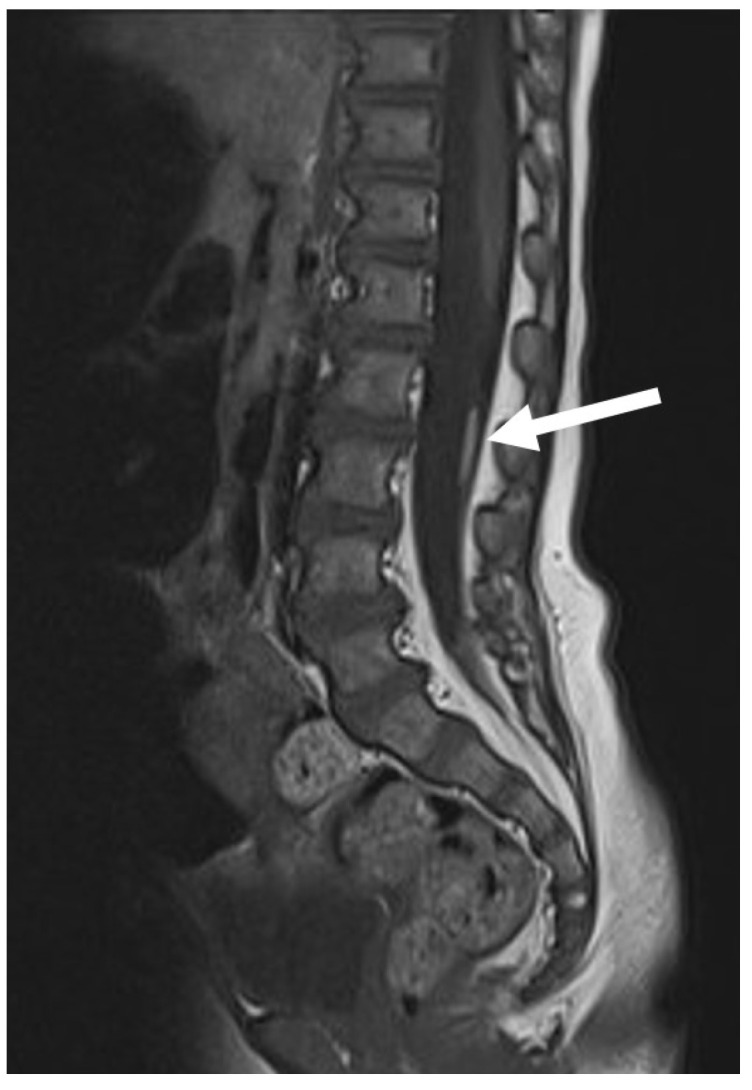
MR appearance of a lipoma of the filum terminale (white arrow), also known as “thickened filum” or “fatty filum”.

1. Lipomas of the filum terminale:

Inadequate apoptosis and regression of the caudal cell mass can lead to a thickened, “fatty” filum terminale. On MRI, the LFT appears as a thick, hyperintensive tract on T1 images (Figure 11). The surgical procedure for untethering is relatively simple: the LFT must be exposed distal to the conus and cut under neurophysiological monitoring. As the risks associated with this procedure are very low, it is worth performing it as early as possible, i.e., in the first months of life [10], in order to protect the child from the onset of symptoms of tethered cord syndrome. In our series, LFTs represented 12/64 (18.7%) of all OSDs and were surgically sectioned in all cases.

2. Currarino syndrome:

Currarino syndrome is characterised by a triad: anal malformation, presacral mass and dysgenesis (or agenesis) of the sacrum. In the case of an associated LFT or conus lipoma, a tethered cord may be present [11]. The presacral mass is, in most cases, a meningocele (Figure 3), but can also represent a teratoma [10]. In the absence of a tethered cord, a conservative approach may be considered if the radiological diagnosis of a presacral meningocele is clear and there is no suspicion of a presacral teratoma.

3. Caudal regression syndrome:

If the process of secondary neurulation does not occur at all, there is complete agenesis of the conus and nerve roots caudal to the S2 level (Figure 4). These patients may have more or less impaired lower limb function, but some can walk normally. Sphincter function is completely absent, as the innervation of these segments is completely undeveloped. Surgical treatment is not indicated in these patients since the conus is absent, but not tethered.

4. Myelocystocele:

Myelocystocele is a cystic malformation that occurs within the developing conus and other neural structures that arise through the process of secondary neurulation. These lesions appear as large cystic formations in the sacral and perineal region. The aim of surgical treatment is to reconstruct the caudal segments of the spinal canal, the dural sac and the anatomy of the pelvic floor itself, which is always severely affected.

### 4.4. Indication for Treatment

OSD are malformations that are specific to the neurosurgical field and for which a neurosurgical opinion should always be sought. Any form of OSD can lead to spinal cord tethering and is therefore a risk factor for progressive neurological symptoms. In this context, the neurosurgeon must always assess whether it is possible to release the spinal cord tethering by surgery. When determining the indication for an operation, the risks of the procedure itself must always be taken into account, in particular the possibility of neurological impairment as a result of the surgical procedure itself.

In most cases, the diagnosis is made in early childhood on the basis of cutaneous stigmata, and the children are usually neurologically intact at birth. If the imaging diagnosis reveals a simple form of OSD, e.g., an LFT, the beneficial effect of surgery is significantly greater compared to a conservative approach. The diagnosis of a transitional or chaotic conus lipoma raises more difficult questions about potential neurological complications associated with surgery, which may be unacceptable to many parents. However, it is becoming increasingly clear in the literature that it is better to operate on the child than to observe them [1,2,8].

In the adult population, the diagnosis of OSD is usually made on the basis of signs and symptoms of neurological deterioration rather than skin manifestations. This can be clearly seen in Table 3, where symptoms of tethered cord syndrome are much more common in adult patients than in children (*p* = 0.0098). Among the symptoms of tethered cord syndrome, pain (*p* = 0.0001) and paraesthesia in the lower limbs (*p* = 0.007) showed the greatest and significant differences between the paediatric and adult populations. Most cases, both paediatric and adult, present with sphincter dysfunction or paresis of the lower limbs. As neurological problems are usually irreversible once they have occurred, it is generally assumed that the protective effect of surgery is less pronounced in adults than in children [1,8]. Nevertheless, certain problems, particularly pain, paresis and paraesthesia, can disappear or at least be alleviated after successful surgical treatment. In particular, it is possible to halt progressive neurological deterioration with surgical treatment and thus prevent further neurological deficits [3].

Based on our results, we have to ask ourselves whether a 44.4% prevalence of permanent neurological deficits after the surgery of complex (transitional and chaotic) conus lipomas is an acceptable fact for parents, compared to a 40–43% chance of having neurological deficits due to the natural history of these lesions [3]. We must underline that more than half of these neurological deficits were represented by isolated dermatomal loss of sensorial function, which, despite being troublesome for the patients, still remains compatible with normal life, normal gait pattern and normal urological function. Statistical models are increasingly in favour of surgery [8], but robust and convincing results can only be based on long-term follow-ups and the long surgical learning curve required for the surgical treatment of the most complex forms of lipomas and, in general, OSD.

## 5. Conclusions

The treatment of OSD requires proper knowledge of the defects that occur during embryological development and can lead to complex malformations. Surgical treatment is always indicated if the OSD constricts the spinal cord below the L1 level and if the malformation itself has a compressive effect on the surrounding nerve tissue. If spinal cord tethering is detected radiologically, it is advisable to perform the untethering as early as possible, to avoid neurological damage caused by spinal cord tension. In our series, the preoperative presence of symptoms was statistically higher in adults than in children (*p* = 0.0098). Surgery of complex spinal cord lipomas was statistically related to a higher rate of postoperative neurological complications (*p* = 0.0002). Such procedures should be carried out in facilities that have the relevant experience and where neurophysiological monitoring is available at all times. After the untethering procedure, children should be monitored throughout their lives, as tethered cord syndrome may recur due to scarring, other associated dysraphic lesions or the incomplete removal of the primary OSD.

## Figures and Tables

**Table 1:** Demographic characteristics of the patients included in the case series.

	Total	Children	Adults
Total number of patients	52	37/52 (71.8%)	15/52 (28.8%)
Total number of OSDs	64	49/64 (76.5%)	15/64 (23.4%)
Cutaneous stigmata	44/52 (84.6%)	34/37 (91.8%)	10/15 (66.6%)
Conus lipoma	22/64 (34.3%)	17/22 (77.2%)	5/22 (22.7%)
Dorsal	13/22 (59%)	12/13 (92.3%)	1/13 (7.6%)
Transitional	7/22 (31.8%)	3/7 (42.8%)	4/7 (57.1%)
Chaotic	2/22 (9%)	2/2 (100%)	0
Dermal tract	13/64 (20.3%)	12/13 (83.3%)	1/13 (7.6%)
LFT	12/64 (18.7%)	10/12 (83.3%)	2/12 (16.6%)
SCM	5/64 (7.8%)	4/5 (80%)	1/5 (20%)
Conus agenesis	3/64 (4.6%)	3/3 (100%)	0
LDM	2/64 (3.1%)	2/2 (100%)	0
Dermoid cyst	2/64 (3.1%)	2/2 (100%)	0
Presacral meningocele	2/64 (3.1%)	0	2/2 (100%)
Neurenteric cyst	1/64 (1.5%)	0	1/1 (100%)
Epithelialized MMC	1/64 (1.5%)	0	1/1 (%)
Meningocele	1/64 (1.5%)	1/1 (100%)	0

**Table 2:** Treatment strategies for the treatment of OSD and complications after surgical treatment.

	Total Number of Patients	Children	Adults
Conservative treatment	17/52 (32.6%)	10/17 (58.8%)	7/17 (41.1%)
Surgical treatment	35/52 (67.3%)	29/35 (82.8%)	6/35 (17.1%)
Transient sphincter dysfunction	2/35 (5.7%)	2/2 (100%)	0
Permanent sphincter dysfunction	1/35 (2.8%)	1/1 (100%)	0
Segmental sensory loss	3/35 (8.5%)	3/3 (100%)	0

**Table 3:** Preoperative signs and symptoms of the tethered cord syndrome recorded in adult and paediatric patients of our series.

	Total	Children	Adults	*t*-Test
Spinal deformities	8/52 (15.3%)	5/37 (13.5%)	3/15 (20%)	*p* = 0.566
Urological problems	12/52 (23%)	7/37 (18.9%)	5/15 (33.3%)	*p* = 0.272
Orthopaedic deformities	17/52 (32.6%)	9/37 (24.3%)	8/15 (53.3%)	*p* = 0.044
Lower limb paresis	15/52 (28.8%)	8/37 (21.6%)	7/15 (46.6%)	*p* = 0.073
Pain	16/52 (30.7%)	5/37 (13.5%)	11/15 (73.3%)	*p* = 0.0001
Paraesthesia	20/52 (38.4%)	10/37 (27%)	10/15 (66.6%)	*p* = 0.007

## Data Availability

No new data were created or analyzed in this study. Data sharing is not applicable to this article.
